# Prevalence, Conversion and Reversion of Tuberculosis Infection Among Healthcare Workers of Tertiary Care Centres in Puducherry, India: A Prospective Cohort Study

**DOI:** 10.3390/tropicalmed10050141

**Published:** 2025-05-20

**Authors:** Sadhana Subramanian, Palanivel Chinnakali, Senbagavalli Prakashbabu, Guha Nandhini Visvanadane, Manju Rajaram, Vijay Viswanathan, Sonali Sarkar, Charles Robert Horsburgh

**Affiliations:** 1 Department of Preventive and Social Medicine, Jawaharlal Institute of Postgraduate Medical Education and Research (JIPMER), Puducherry 605006, India; sadhanasubramanian03@gmail.com (S.S.); prco.indoustb@gmail.com (S.P.); sarkarsonaligh@gmail.com (S.S.); 2 National Tuberculosis Elimination Programme, Puducherry State Health Mission, Puducherry 605006, India; guhnandy@gmail.com; 3 Department of Pulmonary Medicine, Jawaharlal Institute of Postgraduate Medical Education and Research (JIPMER), Puducherry 605006, India; mail2manju22@gmail.com; 4 Prof. M. Viswanathan Diabetes Research Centre, Chennai 600013, India; drvijay@mvdiabetes.com; 5 Department of Epidemiology, Biostatistics, Global Health and Medicine, Boston University, Boston, MA 00215, USA; rhorsbu@bu.edu

**Keywords:** serial testing, latent tuberculosis infection, healthcare professionals

## Abstract

Healthcare workers (HCWs) face an increased risk of tuberculosis (TB) due to occupational exposure. This study aimed to estimate the point prevalence of TB infection (TBI) from the initial test performed, while the reversion and conversion were done by subsequent testing at one year among HCWs in Puducherry, India. A prospective cohort study was conducted among a sample of proportionately chosen HCWs based on their occupational strata of a tertiary hospital in 2022. TBI was assessed using IGRA (4th generation QuantiFeron—TB gold plus kits) after TB symptom screening. The IGRA test was repeated at the end of one year. Reversion was defined as a positive IGRA test at the baseline and had values < 0.2 IU/L in TB1 or TB2 tubes during follow-up. Conversion was defined as a negative IGRA result at the baseline and had values of >0.7 IU/L in TB1 or TB2 tubes during follow-up. Of the 400 HCWs included, the mean (SD) age was 37 (7) years. Median (IQR) work experience was 15.7 (10–21) years. TBI was seen in 150 HCWs (37.7%, 95% CI: 33.0–42.7), and one had active TB. A total of 128/150 HCWs with TBI at baseline were followed up, and 15 had TBI reversion (11.7 per 100 person-years; 95% CI: 6.7–18.5). Thirteen HCWs (5.6 per 100 person-years; 95% CI: 3.3–9.8) had TBI conversion.

## 1. Introduction

Globally, tuberculosis (TB) remains a major public health problem. A recent systematic review reports that about one-fourth (24.8%) of the population is infected with TB bacilli and has not developed the disease yet [1]. The known risk groups for tuberculosis infection (TBI) are household contacts of TB patients, people living with HIV, and people on dialysis and immunosuppressants. Healthcare workers (HCWs) are identified as a risk group by the World Health Organization (WHO) for systematic testing and treatment for TBI. In India, screening for TBI and treatment for HCWs are still in their nascent stages [2].

HCWs are at an increased risk of developing TBI, especially in countries such as India, due to the high TB burden and suboptimal infection control measures in health facilities. The reported prevalence of TBI among HCWs is 47% globally (high TB burden countries) [3]. A study conducted among 200 medical residents and nursing students from Pune, India, reported a TBI incidence of 26.8 (18.6–37.2) per 100 person-years, and 3% progressed to active TB among those with TBI within one year. Only three HCWs among those with prevalent and incident TBIs had completed taking the Isoniazid Preventive Treatment (IPT) [4].

The Tuberculin Skin Test (TST) and Interferon Gamma Release Assay (IGRA) are common tests for TBI. IGRA has some advantages over TST, as BCG vaccination or any other mycobacterium will not interfere with IGRA results [5]. Not everyone infected with M. tuberculosis will develop active TB, and currently, there are no definitive tests to predict disease progression [6].

Due to the variability in T-cell responses to IGRA, there could be variations in the test results, i.e., conversion (negative to positive) or reversion (positive to negative), during sequential testing of IGRA. Recent evidence suggests that IGRA responses could be highly transient (conversion (46%), and reversion (20%)) over a period of time [7]. The variation in the result could be due to the samples being collected and processed during the dormant phase, where TB bacilli might not or intermittently release early secretory antigenic 6 kDa (ESAT-6) and culture filtrate protein 10 (CFP-10) [8].

HCWs in tertiary care settings are exposed to a large number of patients with severe forms of TB that require hospitalization; hence, there is a higher probability of transmission within the hospital. However, routine annual screening for TB disease or TBI in HCWs is not implemented in India. Data from this study can aid in framing policy changes regarding the screening and treatment approach for HCWs. This study aims to determine the prevalence, conversion, and reversion of TBI status among HCWs after a one-year follow-up and factors associated with TBI in a tertiary care institute in Puducherry, South India.

## 2. Materials and Methods

Study design: Prospective cohort study. The recruitment and initial IGRA testing of the participants lasted one year (2022–2023), and conversion and reversion were assessed again at the end of one year (2023–2024).

Study Setting: This study was conducted among healthcare workers (HCWs) in a tertiary care institute in Puducherry, South India. India has an annual TB notification rate of 168 cases per lakh population [9]. The standard diagnostic procedure for tuberculosis involves screening all presumptive TB patients through a sputum smear examination and a chest X-ray, followed by a Cartridge Nucleic Acid Amplification Test (CBNAAT) [10].

This teaching hospital in Puducherry, South India, has about 230 doctors, 1400 nurses, and 100 laboratory technicians. Over 1200 TB patients are diagnosed annually. TBI testing and treatment are done only for people undergoing dialysis and those on immunosuppressants. Six months of daily isoniazid and 3HP (once weekly isoniazid and rifapentine for three months) are the treatment regimens currently available in the institute.

Study population: The study included doctors, nurses, and laboratory technicians (regular employees). HCWs without active TB disease and who were not pregnant during the enrollment were included.

Sample Size Calculation: Using OpenEpi Version 3.01, the sample size was estimated as 400, assuming the prevalence of TBI among HCWs to be 47% in high TB burden countries, 5% absolute precision, 5% alpha error, and 5% non-response rate.

Sampling Technique: A proportionate stratified simple random sampling technique was adopted to select the study participants. Based on the occupation, HCWs were chosen proportionately; 80% represented the nursing fraternity, 14% were physicians/surgeons, and 6% were laboratory technicians.

Study Procedure: Information on socio-demographic characteristics (age, gender, occupation, work experience), behavioral characteristics (alcohol use in the last year, tobacco use ever and in the last month), and history of Diabetes Mellitus (DM) and information related to TB (past history, family history of TB, presence of BCG scar, number of times HCWs had come in contact with known TB patients in the past year) were obtained during the face-to-face interviews.

Anthropometric measurements such as height and weight were recorded. The Asian classification for body mass index was used [11]. About 6 mL of venous blood sample was collected for Interferon-Gamma Release Assay (IGRA) and for glycosylated hemoglobin (HbA1c) testing. Fourth-generation QuantiFERON^®^—TB Gold Plus (QFT-Plus) kits (QIAGEN, Hilden, Germany) were used for TBI testing, and the High-Performance Liquid Chromatography (HPLC) method was used for HbA1c assessment. A trained laboratory technician did IGRA testing at the TB laboratory in the Department of Preventive and Social Medicine. HCWs without symptoms of TB and who tested positive for IGRA were evaluated (chest radiography) for active TB, and a final diagnosis of TBI was made after ruling out active TB by a chest physician. All the HCWs diagnosed with TBI who were willing to take Tuberculosis Preventive Treatment (TPT) were offered six months of daily isoniazid (6INH). If an HCW was identified with active TB, they were excluded from the analysis. At the end of one year, IGRA was repeated for all study participants, along with the behavioral characteristics and clinical history related to TB.

Operational Definition:

TB infection: Values > 0.35 in TB1 tube minus nil tube and >25% of nil value in TB1 or TB2 tubes are TBI positive. TBI negative if the values are <0.35 in the TB1 tube minus the nil tube or >0.35 and <25% of the nil tube value and >0.5 in the mitogen tube (positive control). Values will be <8.0 in nil tubes for MTB-positive and -negative samples, and the unit of measurement is IU/mL. Results are classified as indeterminate if the values are >8.0 in the nil tube and <0.5 in the mitogen minus the nil tube [12].

Conversion: Among those who were negative (<0.35 IU/mL) at the baseline, we used two definitions for conversion. One was based on the cut-off IGRA values of >0.70 IU/mL in TB1 or TB2 tubes, and the other was a positive IGRA result (>0.35 IU/mL in TB1 or TB2 tubes) [13].

Reversion: Among those who were positive for TBI (≥0.35 IU/mL) at the baseline, we used two definitions for reversion. One was based on the cut-off IGRA values of <0.20 IU/mL in TB1 or TB2 tubes, and the other was a negative IGRA result (<0.35 IU/mL in TB1 or TB2 tubes) [13].

Two definitions were used to describe conversion and reversion based on previous literature to account for variability around IGRA thresholds and improve specificity and sensitivity.

Statistical analysis: Data was entered in the REDCap portal, and the analysis was carried out in Stata software version 14 (StataCorp., College Station, TX, USA). The prevalence, conversion, and reversion of TBI were summarized as frequency and percentage with a 95% confidence interval (CI). The true prevalence was accounted for by using the formula (apparent prevalence + specificity-1) (sensitivity + specificity-1). The sensitivity and specificity of the QFT-plus tube are 94% and 97%, respectively [14]. A chi-squared test was performed to find the factors associated with the prevalence, conversion, and reversion of TBI. A *p*-value of less than 0.05 was considered statistically significant. To find the factors associated with the prevalence of TBI, we included all the variables having a *p*-value < 0.2 in unadjusted analysis in the multivariable model (log-binomial regression-family (binomial, link–log; stata command-glm)), after checking for multicollinearity (VIF < 2) and assessment of model fit via deviance and residual plots. An adjusted prevalence ratio with 95% CI was reported.

Ethics and dissemination: The study protocol was reviewed and approved by the Institute Ethics Committee of the tertiary care hospital (approval number: JIP/IEC/2021/330).

## 3. Results

A total of 452 HCWs were approached, of which 400 participants were enrolled, with a response rate of 88%. Non-responders were predominantly physicians compared to nurses and laboratory technicians. Among the 36 HCWs lost to follow-up, a higher proportion were married (72%) and aged 31–45 years (47%). However, no other significant differences were observed in the education status, occupation, or conditions such as DM. The mean (SD) age of the HCWs was 37 (7) years. Most HCWs were nurses (83%) and females (70%). Median (IQR) work experience was 15.7 (10–21) years. Three-fourths (75%) of the HCWs had BCG scars, and 92% of the HCWs had contact with a known TB patient in the past. Sixty-five (16%) HCWs had diabetes mellitus, and 126 (31.5%) had pre-diabetes.

Of the 400 HCWs enrolled, 151 were positive upon IGRA testing at baseline, and none had indeterminate results. Of 151 IGRA positives, one HCW was diagnosed with extra-pulmonary TB (lymph node), and 150/399 had TBI (37.7%; 95% CI: 33.0–42.7). However, the true prevalence is found to be 38.1% (95% CI: 33.5–42.9). Age group > 45 years, work experience >10 years, pre-diabetes, and family history of TB were significantly associated with TBI in univariate analysis. After adjusting for age, gender, work experience, BMI, presence of BCG scar, and family history of TB, none of the associations were statistically significant (*p* > 0.05) (Table 1).

**Figure 1:**
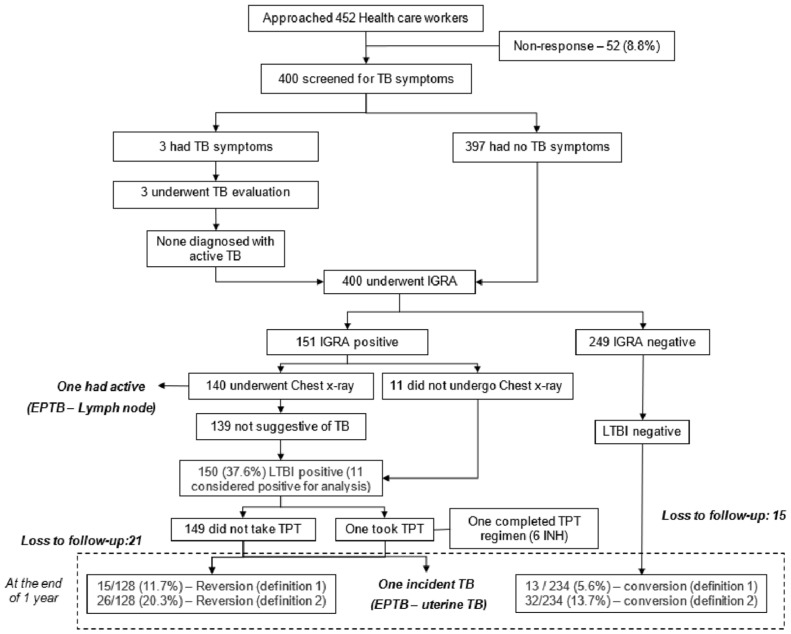
Screening cascade to detect TB infection (TBI), reversion, and conversion among healthcare workers (N = 400).

Of the 150 HCWs, one was willing to take TPT (6 INH) and had completed the regimen, and one was detected with TB disease during the follow-up period. The reasons for TPT refusal are published elsewhere; the most common reasons mentioned are the low perceived threat for progression of TBI to TB disease, the adverse drug reaction, longer treatment duration, and insufficient evidence to say TPT is effective among this group [15]. Follow-up testing at one year was taken up by 128/150 (85.3%) HCWs. The reversion was seen in 15/128 HCWs (11.7 person-years; 95% CI: 6.7–18.5) and 26/128 (20.3 person-years; 95% CI: 13.7–28.3) by definitions 1 and 2, respectively. Of the 249 HCWs negative for TBI at baseline, 234 underwent follow-up testing. Conversion of TBI was observed in 13/234 (5.6 person-years; 95% CI: 3.3–9.8) and 32/234 HCWs (13.7 person-years; 95% CI: 9.5–18.7) by definitions 1 and 2, respectively, over a period of one year (Figure 1).

The reversion of TBI was found to be significantly higher among doctors (41.7%; RR: 4.29; 95% CI: 1.75–10.46) as compared to nurses (9.7%) and laboratory technicians (0%) (Table 2). The incidence of TBI was found to be higher among females, aged between 18 and 30 years, HCWs with >10 years of work experience, those who were underweight, had a BCG scar, had DM, and those who had encountered TB patients, as compared to their counterparts. However, none of the differences were statistically significant (Table 3).

## 4. Discussion

More than one-third of the HCWs had TBI. The reversion was seen among one in ten HCWs, and one in 20 HCWs had conversion within one year. Those aged > 45 years, having work experience of more than ten years, having a BCG scar, having a family history of TB, or having pre-diabetes had a higher prevalence of LTBI, yet this was not statistically significant.

In India, the prevalence of TBI is relatively higher among HCWs as compared to the general population (21.7%; 95% CI: 19.4%–23.9%), and they are considered a high-risk group by the WHO [16,17]. In countries with a high TB burden, the odds of an HCW having TBI were 2.27 (95% CI: 1.67–5.19) times higher, as compared to the general population [18]. There are very few studies from India among HCWs, most of them being done among medical or nursing students. The reported prevalence of TBI varied between 30% and 50%, with a maximum sample size of 200. Few of these studies have reported that increasing age and years of work exposure were significantly associated with a higher prevalence of TBI [4,19,20,21]. In our study, certain factors such as older age, longer work experience, presence of BCG scar, and pre-diabetes showed a trend towards association with TBI, though it was not statistically significant. This may be attributed to the limited sample size for subgroup analyses, which may have reduced the power to detect true associations. Hence, the possibility of a Type II error cannot be ruled out. Future studies with larger cohorts and adequately powered subgroup analyses are warranted to explore these associations more definitively.

Conversion rates ranged between 2% and 15%, while reversion rates ranged between 10% and 45% among HCWs. Studies from low TB-burden countries such as Germany and the U.S.A. report lower conversions (1.9% and 3.2%, respectively) and higher reversions (33.3% and 45%, respectively) using the definition of a change from a negative to positive LTBI test or vice versa [22,23]. The lower TB prevalence and the use of personal protective measures in the countries could be the potential reasons for the varied findings. A study by Pai et al. in India showed conversion (11.6%) and reversion rates (7.5%) similar to our findings. However, when using TST, the conversion rates were only 4%, according to the definition of an increase in TST induration by 10mm [22,23,24,25]. Conversion ranged between 7.5% to 22%, and reversion ranged between 6% and 33% across other high-risk groups such as PLHIV, household contacts, rheumatic diseases, and transplant patients [26,27,28,29]. This may be attributed to the tests adopted (IGRA/TST) for TBI detection, the definition used (change from positive to negative or vice versa or the defined cut-offs) for conversion and reversion, the frequency of testing (6 months/one year/two years), and the population characteristics, and, hence, may not be directly comparable.

Though IGRAs perform relatively better in terms of specificity than TST, they are not considered the gold standard due to the high assay variability, leading to higher conversion and reversion rates. The Interferon-gamma levels vary at random, regardless of the treatment. This could be because of the fluctuation/variation in the immune response exhibited by the individual or due to the procedures followed during sample collection and processing. Hence, all conversions may not have resulted from a new infection [24,30,31].

In high TB burden countries such as India, despite the program’s recommendation for screening and treating HCWs for TBI, it has not been implemented yet [32]. The HCWs have repeated exposure to TB patients, especially in an overcrowded tertiary care setting where they experience severe forms of TB (occupational risk). Annual screening for TB disease and administration of TPT for those with TBI may be considered for HCWs. Recent advancements in TBI diagnostics, which have the advantages of IGRA and TST, like Cy-TB, can be explored for surveillance amongst HCWs [33]. As IGRA is expensive and has a low predictive ability to identify the progression from infection to disease, developing cost-effective and accurate diagnostic tests for TBI detection should be encouraged. In addition, there is a need for the invention of biomarkers that help distinguish between prior exposures treated for TBI and new exposures among HCWs.

Risk factors must be considered before initiating Tuberculosis Preventive Treatment (TPT) in this group. A tool developed by Menzies et al. has accounted for various risk factors (age, DM, co-morbidities, BCG status, any recent contact with TB patients, and country of birth) that state the probability of an individual having TB in the future [34]. Periodic testing for TBI and an annual update of the risk factors mentioned above help the physician decide on initiating TPT. In addition to administering TPT, compliance with certain infection control measures, such as mandating the use of masks by presumptive TB patients, respirators by the HCWs within the hospital campus, and proper crowd management in the OPD area, will reduce transmission in the healthcare setting. Periodical environmental audits to assess the CO_2_ levels will aid us in crowd management at the OPDs, which will, in turn, impact the reduction of TB incidence [35,36,37]. Also, specific engineering control measures such as improved ventilation and UV sterilization, particularly in high-risk clinical areas, could also limit the infection.

Strengths and limitations: A representative sample of HCWs was chosen for this study using stratified random sampling. The techniques for estimating blood glucose levels (HbA1c) and detecting TBI (IGRA) are the most valid measures currently available. The study was not powered enough to determine the factors associated with TBI, as the sample size was primarily calculated to determine the prevalence of TBI. Social desirability bias cannot be ruled out while evaluating alcohol and tobacco usage. We relied upon the self-reported data to determine the extent of TB exposure.

## 5. Conclusions

More than one-third of the HCWs had LTBI at baseline. A one-year follow-up revealed that one in ten exhibited reversions of IGRA, suggesting potential immune clearance. About one in 20 had a conversion of IGRA, highlighting the persistent TB exposure to the HCWs. These findings underscore the critical need for comprehensive LTBI screening and targeted preventive measures for HCWs in high TB burden settings such as India.

## Figures and Tables

**Table 1:** Socio-demographic, behavioral, and clinical profile associated with tuberculosis infection among healthcare workers (N = 399) ^a^.

Variables	Categories	Total	TBI n (%)	cPR (95% CI) *	aPR (95% CI) **	*p*-Value
	Total	399	150 (37.6)	-		-
**Gender**	Male	118	44 (37.3)	1	1	-
	Female	281	106 (37.7)	1.01 (0.77–1.33)	0.93 (0.70–1.23)	0.630
**Age group (in years)**	18–30	47	15 (31.9)	1	1	-
	31–45	278	95 (34.2)	1.07 (0.68–1.68)	0.86 (0.51–1.44)	0.565
	46–58	74	40 (54.1)	1.69 (1.06–2.70)	1.32 (0.73–2.41)	0.348
**Occupation**	Doctor	41	15 (36.6)	1	-	-
	Nurse	329	122 (37.1)	1.01 (0.66–1.55)	-	-
	Laboratory technician	29	13 (44.8)	1.22 (0.69–2.17)	-	-
**Work experience**	≤10 years	126	36 (28.6)	1	1	-
	>10 years	273	114 (41.8)	1.46 (1.07–1.99)	1.32 (0.91–1.93)	0.138
**Tobacco use**	Yes	5	2 (40.0)	1.06 (0.36–3.13)	-	-
	No	394	148 (37.7)	1	-	-
**Alcohol use**	Yes	30	13 (43.3)	1.16 (0.65–1.70)	-	-
	No	369	137 (37.1)	1	-	-
**Body mass index (kg/m^2^)**	Underweight (<18.5)	11	0 (0)	**-**		**-**
	Normal (18.5–22.9)	81	26 (32.1)	1	1	-
	Overweight (23–24.9)	79	34 (43.0)	1.34 (0.89–2.01)	1.31 (0.88–1.97)	0.174
	Obese (≥25)	228	90 (39.5)	1.23 (0.86–1.75)	1.19 (0.83–1.71)	0.335
BCG scar *	Absent	98	42 (42.9)	1.19 (0.91–1.57)	**1.32 (1.00–1.74)**	**0.049**
	Present	301	108 (35.9)	1	1	-
Contact with a TB patient *	Yes	367	139 (37.9)	1.10 (0.67–1.81)	-	-
	No	32	11 (34.4)	1	-	-
Family history of TB *	Yes	71	35 (49.3)	1.41 (1.06–1.86)	1.24 (0.94–1.64)	0.123
	No	328	115 (35.1)	1	1	-
**HbA1c categories (%)**	Diabetes (≥6.5)	65	20 (30.8)	0.89 (0.59–1.33)	0.67 (0.43–1.02)	0.064
	Pre-diabetes (5.6–6.4)	126	58 (46.0)	1.33 (1.02–1.73)	1.16 (0.90–1.51)	0.269
	No diabetes (<5.6)	208	72 (34.6)	1	1	-

* TBI: Tuberculosis infection; TB: Tuberculosis; BCG: Bacillus Calmette-Guérin; cPR: Crude prevalence ratio; aPR: Adjusted prevalence ratio; CI: Confidence interval. ** Adjusted for age, gender, years of work experience, diabetes, body mass index, BCG scar, and family history of tuberculosis. ^a^ One was excluded from the analysis as the HCW was diagnosed with TB.

**Table 2:** Socio-demographic, behavioral, and clinical profile associated with reversion among healthcare workers (N = 128).

Variables	Categories	Total	Operational Definition -1		Operational Definition -2	
			Reversion n (%)	RR (95% CI) *	Reversion n (%)	RR (95% CI) *
	Total	128	15 (11.7)	-	26 (20.3)	-
**Gender**	Male	39	7 (17.9)	2.00 (0.78–5.12)	11 (28.2)	1.67 (0.85–3.30)
	Female	89	8 (9.0)	1	15 (16.8)	1
**Age group (in years)**	18–30	11	0 (0)	-	1 (9.1)	1
	31–45	84	11 (13.1)	1.08 (0.37–3.15)	16 (19.1)	2.09 (0.31–14.29)
	46–58	33	4 (12.1)	1	9 (27.3)	3.00 (0.43–21.09)
**Occupation**	Doctor	12	5 (41.7)	4.29 (1.75–10.46)	7 (58.3)	3.79 (0.97–14.80)
	Nurse	103	10 (9.7)	1	17 (16.5)	1.07 (0.28–4.12)
	Laboratory technician	13	0 (0)	-	2 (15.4)	1
**Work experience**	≤10 years	28	2 (7.1)	1	4 (14.3)	1
	>10 years	100	13 (13.0)	1.82 (0.44–7.60)	22 (22.0)	1.54 (0.58–4.10)
**Body mass index (kg/m^2^)**	Underweight (<18.5)	0	0 (0)	-	0	-
	Normal (18.5–22.9)	21	0 (0)	-	3 (14.3)	1
	Overweight (23–24.9)	28	4 (14.3)	1.02 (0.35–2.96)	8 (28.6)	2.00 (0.60–6.64)
	Obese (≥25)	79	11 (13.9)	1	15 (19.0)	1.33 (0.42–4.16)
BCG scar *	Absent	37	6 (16.2)	1.63 (0.63–4.28)	10 (27.0)	1.54 (0.77–3.07)
	Present	91	9 (9.9)	1	16 (17.6)	1
Contact with a TB patient *	Yes	119	14 (11.8)	1	24 (20.2)	1
	No	9	1 (11.1)	0.94 (0.13–6.39)	2 (22.2)	0.90 (0.25–3.24)
Family history of TB *	Yes	30	5 (16.7)	1.63 (0.60–4.40)	7 (23.3)	1.20 (0.56–2.58)
	No	98	10 (10.2)	1	19 (19.4)	1
**HbA1c categories (%)**	Diabetes (≥6.5)	21	0 (0)	-	3 (14.3)	1
	Pre-diabetes (5.6–6.4)	36	4 (11.1)	1	7 (19.4)	1.36 (0.39–4.70)
	No diabetes (<5.6)	71	11 (15.5)	1.39 (0.48–4.07)	16 (22.5)	1.58 (0.51–4.90)

TB: Tuberculosis; BCG: Bacillus Calmette-Guérin; RR—Risk ratio; CI—Confidence Interval. * Reversion: ≥0.35 IU/mL at baseline and cut-off IGRA values of < 0.20 IU/mL in TB1 or TB2 tubes (operational definition 1) or a negative IGRA result with < 0.35 IU/mL in TB1 or TB2 tubes (operational definition 2).

**Table 3:** Socio-demographic, behavioral, and clinical profile associated with conversion among healthcare workers (N = 234) *.

Variables	Categories	Total	Operational Definition -1		Operational Definition -2	
			Conversion n (%)	RR (95% CI) *	Conversion n (%)	RR (95% CI) *
	Total	234	13 (5.6)	-	32 (13.7)	-
**Gender**	Male	71	1 (1.4)	1	5 (7.0)	1
	Female	163	12 (7.4)	5.22 (0.69–39.43)	27 (16.6)	2.40 (0.94–5.85)
**Age group (in years)**	18–30	26	2 (7.7)	2.46 (0.24–25.64)	4 (15.4)	1.64 (0.40–6.68)
	31–45	176	10 (5.7)	1.81 (0.24–13.71)	25 (14.2)	1.51 (0.49–4.72)
	46–58	32	1 (3.1)	1	3 (9.4)	1
**Occupation**	Doctor	25	0 (0.0)	-	1 (4.0)	1
	Nurse	194	13 (6.7)	-	29 (14.9)	3.7 (0.53–26.2)
	Laboratory technician	15	0 (0)	-	2 (13.3)	3.3 (0.33–33.7)
**Work experience**	≤10 years	83	4 (4.9)	1	10 (12.0)	1
	>10 years	151	9 (6.0)	1.24 (0.39- 3.90)	22 (14.6)	1.2 (0.60–2.43)
**Body mass index (kg/m^2^)**	Underweight (<18.5)	10	2 (20.0)	9.60 (0.96–95.91)	2 (20.0)	1.6 (0.38–6.80)
	Normal (18.5–22.9)	48	1 (2.1)	1	6 (12.5)	1
	Overweight (23–24.9)	43	2 (4.6)	2.23 (0.21–23.76)	4 (9.3)	0.7 (0.22–2.46)
	Obese (≥25)	133	8 (6.0)	2.89 (0.37–22.48)	20 (15.0)	1.2 (0.51–2.81)
BCG scar *	Absent	52	2 (3.8)	0.64 (0.15–2.78)	8 (15.4)	1.17 (0.56–2.44)
	Present	182	11 (6.0)	1	24 (13.2)	1
Contact with a TB patient *	Yes	215	12 (5.6)	1.06 (0.14–7.72)	30 (13.9)	1.32 (0.34–5.12)
	No	19	1 (5.3)	1	2 (10.5)	1
Family history of TB *	Yes	35	0 (0.0)	-	2 (5.7)	0.38 (0.09–1.51)
	No	199	13 (6.5)	-	30 (15.1)	1
**HbA1c categories (%)**	Diabetes (≥ 6.5)	46	3 (6.5)	1.20 (0.33–4.33)	7 (15.2)	1.12 (0.50–2.47)
	Pre-diabetes (5.6–6.4)	41	2 (4.9)	0.90 (0.20–4.05)	5 (12.2)	0.90 (0.36–2.24)
	No diabetes (<5.6)	147	8 (5.4)	1	20 (13.6)	1

TB: Tuberculosis; BCG: Bacillus Calmette-Guérin; RR—Risk ratio; CI—Confidence Interval. * Conversion: <0.35 IU/mL at baseline and cut-off IGRA values of >0.70 IU/mL in TB1 or TB2 tubes (operational definition 1) or a positive IGRA result with ≥0.35 IU/mL in TB1 or TB2 tubes (operational definition 2).

## Data Availability

The dataset used for this study is available from the corresponding author upon reasonable request.

## References

[1] Cohen A., Mathiasen V.D., Schön T., Wejse C. (2019). The Global Prevalence of Latent Tuberculosis: A Systematic Review and Meta-Analysis. Eur. Respir. J..

[2] (2019). World Health Organization Consolidated Guidelines on Tuberculosis Treatment.

[3] Nasreen S., Shokoohi M., Malvankar-Mehta M.S. (2016). Prevalence of Latent Tuberculosis among Health Care Workers in High Burden Countries: A Systematic Review and Meta-Analysis. PLoS ONE.

[4] Kinikar A., Chandanwale A., Kadam D., Joshi S., Basavaraj A., Pardeshi G., Girish S., Shelke S., DeLuca A., Dhumal G. (2019). High Risk for Latent Tuberculosis Infection among Medical Residents and Nursing Students in India. PLoS ONE.

[5] Pai M., Zwerling A., Menzies D. (2008). Systematic Review: T-Cell-Based Assays for the Diagnosis of Latent Tuberculosis Infection: An Update. Ann. Intern. Med..

[6] Gill C.M., Dolan L., Piggott L.M., McLaughlin A.M. (2022). New Developments in Tuberculosis Diagnosis and Treatment. Breathe.

[7] Wang M.-S., Li-Hunnam J., Chen Y.-L., Gilmour B., Alene K.A., Zhang Y.-A., Nicol M.P. (2024). Conversion or Reversion of Interferon Gamma Release Assays for Mycobacterium Tuberculosis Infection: A Systematic Review and Meta-Analysis. Clin. Infect. Dis. Off. Publ. Infect. Dis. Soc. Am..

[8] Pai M., O’Brien R. (2007). Serial Testing for Tuberculosis: Can We Make Sense of T Cell Assay Conversions and Reversions?. PLoS Med..

[9] National Tuberculosis Elimination Programme (2021). Central TB Division. India TB Report 2021.

[10] National Tuberculosis Elimination Programme (2020). Training Modules for Programme Managers and Medical Officers.

[11] Centers for Disease Control and Prevention About Adult BMI|Healthy Weight, Nutrition, and Physical Activity|CDC. https://www.cdc.gov/bmi/adult-calculator/bmi-categories.html.

[12] Mazurek G.H., Jereb J., Vernon A., LoBue P., Goldberg S., Castro K. (2010). Updated Guidelines for Using Interferon Gamma Release Assays to Detect Mycobacterium Tuberculosis Infection—United States, 2010. MMWR. Recomm. Rep. Morb. Mortal. Wkly. Rep. Recomm. Rep..

[13] Pai M., Joshi R., Dogra S., Zwerling A.A., Gajalakshmi D., Goswami K., Reddy M.V.R., Kalantri A., Hill P.C., Menzies D. (2009). T-Cell Assay Conversions and Reversions among Household Contacts of Tuberculosis Patients in Rural India. Int. J. Tuberc. Lung Dis. Off. J. Int. Union Against Tuberc. Lung Dis..

[14] Qiagen QuantiFERON-TB Gold Plus (QFT-Plus). https://www.qiagen.com/us/products/diagnostics-and-clinical-research/tb-management/quantiferon-tb-gold-plus-us.

[15] Subramanian S., Gnanadhas J., Sarkar S., Rajaram M., Prakashbabu S., Chinnakali P. (2024). Why Do Healthcare Workers Refuse Tuberculosis Preventive Treatment (TPT)? A Qualitative Study from Puducherry, South India. BMJ Open Respir. Res..

[16] (2018). World Health Organisation Latent Tuberculosis Infection: Updated and Consolidated Guidelines for Programmatic Management.

[17] National TB Elimination Programme (2021). Central TB Division. National TB Prevalence Survey in India 2019–2021.

[18] Uden L., Barber E., Ford N., Cooke G.S. (2017). Risk of Tuberculosis Infection and Disease for Health Care Workers: An Updated Meta-Analysis. Open Forum Infect. Dis..

[19] Christopher D.J., Daley P., Armstrong L., James P., Gupta R., Premkumar B., Michael J.S., Radha V., Zwerling A., Schiller I. (2010). Tuberculosis Infection among Young Nursing Trainees in South India. PLoS ONE.

[20] Dabhi P.A., Thangakunam B., Gupta R., James P., Thomas N., Naik D., Christopher D.J. (2020). Screening for Prevalence of Current TB Disease and Latent TB Infection in Type 2 Diabetes Mellitus Patients Attending a Diabetic Clinic in an Indian Tertiary Care Hospital. PLoS ONE.

[21] Pai M., Gokhale K., Joshi R., Dogra S., Kalantri S., Mendiratta D.K., Narang P., Daley C.L., Granich R.M., Mazurek G.H. (2005). Mycobacterium Tuberculosis Infection in Health Care Workers in Rural India: Comparison of a Whole-Blood Interferon Gamma Assay with Tuberculin Skin Testing. JAMA.

[22] Ringshausen F.C., Nienhaus A., Schablon A., Schlösser S., Schultze-Werninghaus G., Rohde G. (2010). Predictors of Persistently Positive Mycobacterium-Tuberculosis-Specific Interferon-Gamma Responses in the Serial Testing of Health Care Workers. BMC Infect. Dis..

[23] Joshi M., Monson T.P., Joshi A., Woods G.L. (2014). IFN-γ Release Assay Conversions and Reversions. Challenges with Serial Testing in U.S. Health Care Workers. Ann. Am. Thorac. Soc..

[24] Pai M., Joshi R., Dogra S., Mendiratta D.K., Narang P., Kalantri S., Reingold A.L., Colford J.M., Riley L.W., Menzies D. (2006). Serial Testing of Health Care Workers for Tuberculosis Using Interferon-γ Assay. Am. J. Respir. Crit. Care Med..

[25] Joshi R., Reingold A.L., Menzies D., Pai M. (2006). Tuberculosis among Health-Care Workers in Low- and Middle-Income Countries: A Systematic Review. PLoS Med..

[26] Aichelburg M.C., Reiberger T., Breitenecker F., Mandorfer M., Makristathis A., Rieger A. (2014). Reversion and Conversion of Interferon- γ Release Assay Results in HIV-1—Infected Individuals Baseline Characteristics of the Study Participants. J. Infect. Dis..

[27] Pai M., Joshi R., Dogra S., Mendiratta D.K., Narang P., Dheda K., Kalantri S. (2006). Persistently Elevated T Cell Interferon-Gamma Responses after Treatment for Latent Tuberculosis Infection among Health Care Workers in India: A Preliminary Report. J. Occup. Med. Toxicol..

[28] Roth P.J., Grim S.A., Gallitano S., Adams W., Clark N.M., Layden J.E. (2016). Serial Testing for Latent Tuberculosis Infection in Transplant Candidates: A Retrospective Review. Transpl. Infect. Dis. Off. J. Transplant. Soc..

[29] Wigg A.J., Narayana S.K., Anwar S., Ramachandran J., Muller K., Chen J.W., John L., Hissaria P., Kaambwa B., Woodman R.J. (2019). High Rates of Indeterminate Interferon-Gamma Release Assays for the Diagnosis of Latent Tuberculosis Infection in Liver Transplantation Candidates. Transpl. Infect. Dis. Off. J. Transplant. Soc..

[30] van Zyl-Smit R.N., Zwerling A., Dheda K., Pai M. (2010). Within-Subject Variability of Interferon-g Assay Results for Tuberculosis and Boosting Effect of Tuberculin Skin Testing: A Systematic Review. PLoS ONE.

[31] Yoshiyama T., Harada N., Higuchi K., Nakajima Y., Ogata H. (2009). Estimation of Incidence of Tuberculosis Infection in Health-Care Workers Using Repeated Interferon-γ Assays. Epidemiol. Infect..

[32] National TB Elimination Programme (2021). Central TB Division Guidelines for Programmatic Managment of Tuberculosis Preventive Treatment in India.

[33] Aggerbeck H., Ruhwald M., Hoff S.T., Borregaard B., Hellstrom E., Malahleha M., Siebert M., Gani M., Seopela V., Diacon A. (2018). C-Tb Skin Test to Diagnose Mycobacterium Tuberculosis Infection in Children and HIV-Infected Adults: A Phase 3 Trial. PLoS ONE.

[34] Menzies D., Gardiner G., Farhat M., Greenaway C., Pai M. (2008). Thinking in Three Dimensions: A Web-Based Algorithm to Aid the Interpretation of Tuberculin Skin Test Results. Int. J. Tuberc. Lung Dis..

[35] Du C.-R., Wang S.-C., Yu M.-C., Chiu T.-F., Wang J.-Y., Chuang P.-C., Jou R., Chan P.-C., Fang C.-T. (2020). Effect of Ventilation Improvement during a Tuberculosis Outbreak in Underventilated University Buildings. Indoor Air.

[36] Rudnick S.N., Milton D.K. (2003). Risk of Indoor Airborne Infection Transmission Estimated from Carbon Dioxide Concentration. Indoor Air.

[37] Gupta S., Abimbola T., Date A., Suthar A.B., Bennett R., Sangrujee N., Granich R. (2014). Cost-Effectiveness of the Three I’s for HIV/TB and ART to Prevent TB among People Living with HIV. Int. J. Tuberc. Lung Dis..

